# Host Immune Evasion by Lyme and Relapsing Fever Borreliae: Findings to Lead Future Studies for *Borrelia miyamotoi*

**DOI:** 10.3389/fimmu.2017.00012

**Published:** 2017-01-19

**Authors:** Brandee L. Stone, Catherine A. Brissette

**Affiliations:** ^1^Department of Biomedical Sciences, School of Medicine and Health Sciences, University of North Dakota, Grand Forks, ND, USA

**Keywords:** *Borrelia miyamotoi*, spirochetes, relapsing fever, Lyme disease, complement, factor H, antigenic variation, immune response

## Abstract

The emerging pathogen, *Borrelia miyamotoi*, is a relapsing fever spirochete vectored by the same species of *Ixodes* ticks that carry the causative agents of Lyme disease in the US, Europe, and Asia. Symptoms caused by infection with *B. miyamotoi* are similar to a relapsing fever infection. However, *B. miyamotoi* has adapted to different vectors and reservoirs, which could result in unique physiology, including immune evasion mechanisms. Lyme *Borrelia* utilize a combination of *Ixodes*-produced inhibitors and native proteins [i.e., factor H-binding proteins (FHBPs)/complement regulator-acquiring surface proteins, p43, BBK32, BGA66, BGA71, CD59-like protein] to inhibit complement, while some relapsing fever spirochetes use C4b-binding protein and likely *Ornithodoros*-produced inhibitors. To evade the humoral response, *Borrelia* utilize antigenic variation of either outer surface proteins (Osps) and the Vmp-like sequences (Vls) system (Lyme borreliae) or variable membrane proteins (Vmps, relapsing fever borreliae). *B. miyamotoi* possesses putative FHBPs and antigenic variation of Vmps has been demonstrated. This review summarizes and compares the common mechanisms utilized by Lyme and relapsing fever spirochetes, as well as the current state of understanding immune evasion by *B. miyamotoi*.

## Introduction

Tick-borne diseases are among the top reported diseases to the US Centers for Disease Control and Prevention. This group of diseases include an array of viral, bacterial, and parasitic pathogens (e.g., Lyme disease, tick-borne relapsing fever, anaplasmosis, rickettsiosis, Powassan virus, tick-borne encephalitis virus, Colorado tick fever, Heartland virus, babesisosis) transmitted by the bite of certain species of hard and soft shell ticks from four genera (*Ixodes, Dermacentor, Amblyomma, Ornithodoros*) (1–3).

Intense research efforts are occurring worldwide in an attempt to understand, detect, control, treat, and eradicate these pathogens and their diseases. One step toward preventing and treating infectious diseases is to understand how pathogens evade host defenses to establish infection. Pathogenic Lyme and relapsing fever borreliae establish infection through one or more of the following mechanisms: physical barriers (e.g., slime layer of glycoproteins), migration to immunoprivileged sites, and hijacking host processes [e.g., inactivation of complement with factor H-binding proteins (FHBPs)]. The mechanisms utilized by the emerging relapsing fever pathogen, *Borrelia miyamotoi*, are currently uncharacterized. Herein, we review some mechanisms Lyme and relapsing fever *Borrelia* utilize to inhibit and evade host complement and humoral immune responses and relate these to mechanisms that might be used by *B. miyamotoi*.

### Lyme Disease and Tick-Borne Relapsing Fever: Spirochetes, Vectors, and Diseases

Approximately 20 closely related pathogenic and non-pathogenic species of *Borrelia* form the *B. burgdorferi sensu lato* complex. Of these 20 species, at least five are classified as causative agents of Lyme disease (US: *B. burgdorferi sensu stricto*; Europe and Asia: *B. afzelii, B. garinii, B. spielmanii, B. bavariensis*) (4–10). Lyme borreliae are carried and transmitted by several species of *Ixodes* ticks (Ixodidae, hard shell) though the most common species are *I. scapularis* and *I. pacificus* in the US and *I. ricinus* and *I. persulcatus* in Europe and Asia.

Species of *Ornithodoros* ticks (Argasidae, soft shell) carry and transmit relapsing fever spirochetes. Several *Borrelia* spp. cause relapsing fever but *B. hermsii, B. turicatae, B. crocidurae, B. hispanica, B. duttonii* are more commonly encountered.

While the general rule is *Ixodes* transmit spirochetes of the *B. burgdorferi s.l*. complex and *Ornithodoros* transmit relapsing fever borreliae, there are exceptions. *B. recurrentis* is a louse-borne relapsing fever spirochete endemic mainly to sub-Saharan Africa. *B. theileri* causes bovine borreliosis and is transmitted by *Rhipicephalus microplus*, a hard shell tick that parasitizes livestock (11). *B. lonestari* and *B. turcica*, spirochetes genetically similar to relapsing fever borreliae, are found in the hard shell ticks, *Amblyomma americanum* and *Hyalomma aegyptium*, respectively (12, 13). Finally, *B. miyamotoi* is a relapsing fever spirochete vectored by the same *Ixodes* spp. that transmit species of the *B. burgdorferi s.l*. complex.

In terms of disease, several tick-borne diseases are associated with non-specific symptoms (i.e., a possibly self-limiting “influenza-like” illness characterized by malaise, fatigue, aches, fever, and chills) (Table 1). While infection with *Borrelia* spp. generally results in similar symptoms, some species-specific symptoms can arise (14, 15). Erythema migrans and arthritis are commonly associated with a *B. burgdorferi s.s*. infection but rarely with *B. afzelii* infection, which more commonly manifests in the dermatological condition, acrodermatitis chronica atrophicans. *B. garinii* is more commonly associated with neurological symptoms. Relapsing fever is characterized by recurring spirochetemia corresponding to recurrent episodes of high fever not seen with *B. burgdorferi s.l*. infections.

**Table 1:** **Overview of *Borrelia* diseases**.

Disease	Vector^a^	Causative agent(s)^a^	Clinical symptom(s)
Lyme disease	*I. scapularis* (USA) *I. pacificus* (USA) *I. ricinus* (Europe, Asia) *I. persulcatus* (Europe, Asia)	*B. burgdorferi sensu stricto* (USA) *B. afzelii* (Europe, Asia) *B. bavariensis* (Europe, Asia; formerly *B. garinii* OspA serotype 4) *B. garinii* (Europe, Asia) *B. spielmanii* (Europe, Asia)	Symptom onset after exposure: early stage generally 3–30 days Influenza-like (e.g., mild fever, malaise, myalgia/arthralgia; *B. burgdorferi s.s*.) Erythema migrans (*B. burgdorferi s.s*., *B. afzelii*) Symptom onset after exposure: late stage generally >30 days Arthritis Acrodermatitis chronica atrophicans (*B. afzelii*) Neurological (Lyme neuroborreliosis, e.g., numbness, Bell’s palsy, stiffness of neck, declining memory, sleep disorders; *B. burgdorferi s.s*., *B. bavariensis*)
Tick-borne relapsing fever	*O. hermsi* *O. turicata* *O. parkeri* *O. moubata*	*B. hermsiiB. turicatae* *B. parkerii* *B. duttonii*	Symptom onset: ca. 7 days Influenza-like Recurring high fever Headache Myalgia Arthritis Approximately 3–10 febrile episodes (relapses) occur; mortality rates are variable but generally less than 5%
Hard tick-borne relapsing fever/*Borrelia miyamotoi* disease	*I. scapularisI pacificus* *I ricinus* *I. persulcatus*	*B. miyamotoi*	Symptom onset after exposure: ca. 15 days (85) Influenza-like Most common: Fever Malaise Headache Chills Arthritis/arthralgia Meningoencephalitis (immunocompromised patients) Rare (less than 10% of patients): Rash/erythema migrans Gastrointestinal (e.g., vomiting, nausea, diarrhea) Cardiac/respiratory (shortness of breath) Neurological (e.g., dizziness, confusion) Stiffness of neck
Louse-borne relapsing fever	*P. humanus humanus*	*B. recurrentis*	Symptom onset after exposure: ca. 4–8 days Recurring high fever Malaise Headache Chills Meningism Myalgia Nausea Vomiting Approximately 3–5 relapses occur; mortality rate varies greatly (30–70% without treatment during outbreaks)

*^a^Commonly encountered and studied vectors and causative agents are listed*.

### *Borrelia* *miyamotoi*

*Borrelia miyamotoi s.s*. strains were first isolated and cultured in Japan in 1995 from *I. persulcatus* and the blood of *Apodemus argenteus* (small Japanese field mouse) (16). Since this initial isolation, *B. miyamotoi* DNA has been identified in *I. scapularis, I. pacificus, I. ricinus*, and *I. persulcatus* across the Northern hemisphere (17–84). *B. miyamotoi* DNA has also been identified in humans with a suspected tick-borne disease; while *B. miyamotoi* is associated with disease, teasing out the details of an infection with this spirochete has proven difficult for several reasons (85–92).

First, diagnoses based on serology can be problematic and lead to false-negative diagnoses. Several antigens, including 4 of the 10 assayed in a Lyme Western blot, are shared among Lyme, relapsing fever, and *B. miyamotoi* spirochetes (93, 94). Although Lyme and relapsing fever *Borrelia* cause different diseases and occupy different niches, species in this genus share a high degree of genetic homology (95–98). Therefore, some degree of cross-reactivity occurs between *B. miyamotoi* antibodies and *B. burgdorferi s.l*. antigens (91).

Second, an adequate and appropriate immunocompetent animal model to study *B. miyamotoi* infection is only now beginning to take shape. Without an optimal animal model to identify characteristic symptoms and pathologies, we are left to interpret and extrapolate symptoms from complex human cases where disease pathology can be complicated by underlying or unreported medical conditions or coinfections. Previous attempts to infect immunocompetent *Peromyscus leucopus* mice (a common reservoir for *B. burgdorferi* in the US) with *B. miyamotoi s.l*. LB-2001 (US strain) had been unsuccessful leaving severe combined immune deficient (SCID) mice as the only available animal model (17). SCID mice infected with *B. miyamotoi* exhibit sustained spirochetemia, similar to infection with relapsing fever spirochetes (99). Recently, however, Wagemakers et al. (100) were able to successfully infect immunocompetent C3H/HeN mice with LB-2001 and demonstrate spirochetemia 2 days post infection (dpi). Three of the eight mice infected exhibited relapsing spirochetemia at 5 and 6 dpi. More studies are required to determine the optimal animal model for *B. miyamotoi* infection (101–103).

Finally, *B. miyamotoi*’s status as a pathogen has only recently been established. The first confirmed human infections were reported in Russia in 2011 (85) with more cases subsequently described in the US, Europe, and Japan (86–91, 104–107).

#### *B. miyamotoi* Infection and Disease

Much of the data available on *B. miyamotoi* infections come from retrospective serological analyses of banked patient samples, which provide valuable epidemiological information but can lack the detailed patient history or clinical aspects required to sufficiently define a disease. The available data depict an illness, currently termed *B. miyamotoi* disease or hard tick-borne relapsing fever that is similar to relapsing fever.

The patients described by Platonov et al. (85) reported tick bites, developed moderate or severe disease, and were hospitalized as a precautionary measure against more severe tick-borne diseases, particularly viral tick-borne encephalitis. In total, 46 patients were classified as having a confirmed *B. miyamotoi* infection with no detected current *B. burgdorferi s.l*. coinfection by PCR. Sera from all 46 patients reacted with whole cell lysates of *B. burgdorferi, B. afzelii*, and *B. garinii*. The most common symptoms were fever, headache, and malaise or fatigue (Table 2). Five patients reported recurrent fever with an average duration of 3.4 days, and 9 days between relapses, similar to infections with relapsing fever spirochetes. All patients were successfully treated with ceftriaxone or doxycycline.

**Table 2:** **Comparison of symptoms reported from US (91) and Russian (85) patients**.

Symptom	US (*n* = 51)	Russia (*n* = 46)
Fever, chills	96%	98%, 35%^a^
Headache	96%^b^	89%
Myalgia	84%	59%
Arthralgia	76%	28%
Malaise/fatigue	82%	98%
Rash/EM^c^	8%	9%
Gastrointestinal symptoms^d^	6%	30% (nausea)
		7% (vomiting)
Respiratory symptoms^e^	6%	na^f^
Neurological symptoms (dizziness, confusion, vertigo)	8%	na
Stiff neck	na	2%

*^a^Fever and chills were reported in separate categories*.

*^b^Authors noted in most patients the headaches were severe*.

*^c^US patients were described as having a rash. Russian patients were noted for having a single erythema migrans*.

*^d^For US patients, GI symptoms included nausea, abdominal pain, diarrhea, anorexia. For Russian patients, GI symptoms included nausea and vomiting*.

*^e^Labored breathing or short of breath*.

*^f^Not reported*.

A similar series of cases were reported in the US in 2015 (91). Ninety-seven of 11,515 patient samples submitted by clinical laboratories for tick-borne disease analysis were PCR-positive for *B. miyamotoi*. Patients with known or suspected *B. burgdorferi* coinfection or a history of Lyme disease were omitted from further analysis. Fever, headache, and malaise were commonly reported among US patients with two patients reporting recurrent fever (Table 2). The duration of febrile episodes and the time between relapses were not reported. Spirochetemia was noted in US patients but was either not reported or documented in Russian patients. Strikingly, a rash or single erythema migrans of unknown origin was reported in 8 and 9% of US and Russian patients, respectively.

Some symptoms were different between the US and Russia patients, which suggest clinical manifestations vary by *B. miyamotoi* strain, similar to that seen with *B. burgdorferi s.l*. strains (Table 2) (108). Arthralgia was more common in US (76%) compared to Russian patients (28%), and leukopenia, thrombocytopenia, and elevated liver enzymes were found in some US patients but in none of the Russian patients. These differences may be explained by genetic differences between American and Asian type *B. miyamotoi*. Genetic analyses of *B. miyamotoi* isolates have revealed heterogeneity between, and a high degree of homology among, strains from the US (American types; *I. scapularis, I. pacificus*), Europe (European type; *I. ricinus*), and Asia (Asian type; *I. persulcatus*) (59, 109).

Detailed case reports are currently available for nine patients in the US, Europe, and Japan. For immunocompetent patients, symptoms were similar to those observed in the aforementioned studies (e.g., fever, headache, malaise) (86, 89, 90, 106, 107). One US patient did not seek treatment, providing additional evidence that *B. miyamotoi* can result in recurrent fever and be self-resolving, similar to other relapsing fever infections (92, 110). This patient experienced two episodes of fever separated by 3 weeks, significantly longer than in other *B. miyamotoi* or relapsing fever patients, with each episode lasting 4–5 days, on par with *B. miyamotoi* or relapsing fever patients.

The pathology of *B. miyamotoi* infection is dramatically different in immunocompromised patients, specifically those treated for non-Hodgkin’s lymphoma (NHL) with rituximab. Two patients treated with rituximab for NHL, one from the US (88) and one from the Netherlands (87), with reported recent tick bites developed meningoencephalitis. Motile spirochetes were detected in cerebral spinal fluid in both cases. Interestingly, *glpQ* was amplified and sequenced from both patient’s samples yet no anti-GlpQ antibodies were detected in the blood or cerebral spinal fluid of the European patient. IgM against *B. burgdorferi* was negative for both patients. Neither patient reported any of the commonly associated symptoms of a *B. miyamotoi* infection (e.g., fever, headache, myalgia, malaise). Instead, both patients exhibited neurological symptoms (cognitive processing defects, disturbed gait). A third patient from Germany, also treated with rituximab for NHL, developed Lyme neuroborreliosis-like symptoms (dizziness, vomiting, and headache) (111).

## The Complement System

**Figure 1:**
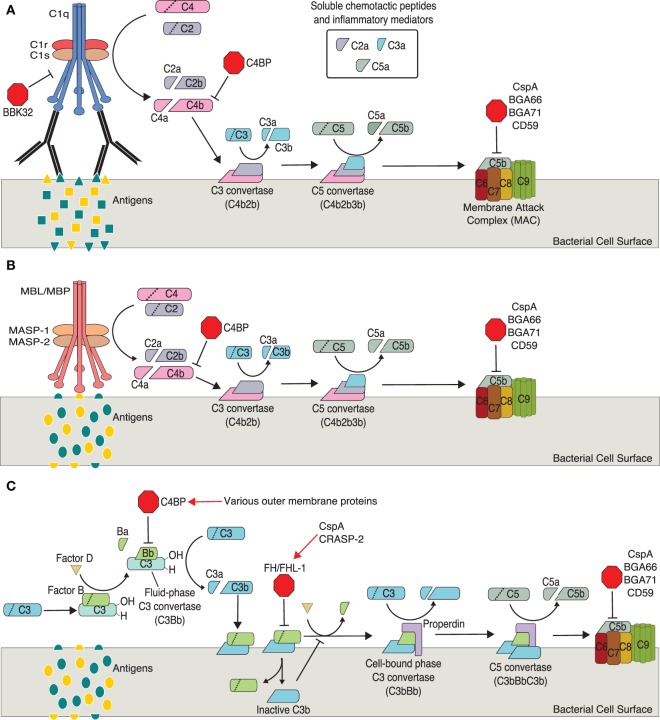
**Activation and regulation of complement pathways relevant to *Borrelia* spp. infection**. **(A)** Classical pathway. **(B)** Mannose–lectin pathway. **(C)** Alternative pathway. Points of complement inhibition utilized by *Borrelia* spp. are indicated by red octagons. Red arrows indicate borrelial proteins that interact with host regulatory proteins.

The complement system, composed of the classical, lectin, and alternative branches, is a crucial component of the immune system (Figure 1). Components of complement continuously circulate in blood making complement one of the first lines of defense against pathogens. Complement initiates an immune response by: (1) triggering phagocytosis through opsonization, (2) mediating inflammation through the release of chemotactic peptides, and (3) lysing cells *via* the membrane attack complex (MAC, also called the terminal complement complex or TCC) (Figure 1) (112).

The classical pathway is generally mediated by non-specific antibodies, immunoglobulin G (IgG) or IgM, binding a bacterial antigen. Importantly, recent studies have shown *Borrelia*-specific IgM is produced by a subset of B cells during infection and plays a crucial role in clearing *Borrelia* (113–121). The C1 complex, composed of C1q, C1r, and C1s, forms upon recognition of bound IgG or IgM. C1 cleaves C2 (C2a, C2b) and C4 (C4a, C4b). C4b covalently binds the target’s cell surface and complexes with C2a to form C3 convertase, which cleaves C3 into C3a and C3b. C3b covalently binds the target cell surface (opsonization, facilitates phagocytosis of foreign cells and cellular debris), while C3a remains soluble to act as a mediator of inflammation. C5 convertase forms when C3b binds C3 convertase. Not surprisingly, C5 convertase cleaves C5 into C5a, a soluble inflammatory mediator, and C5b. C5b binds the target cell surface and C6 forming C5b6, which binds C7 (C5b–7) then C8. The C5b–8 complex binds C9 (C5b–9) and facilitates polymerization of several additional C9 proteins. These polymerized C9 proteins form the transmembrane pore of the MAC allowing an influx of extracellular fluid and subsequent lysis of the target cell.

The lectin pathway is very similar to the classical pathway, differing only in the initiation steps. The lectin pathway is typically initiated through mannose-binding lectins, a group of pattern recognition receptors (PRRs) on host cells, binding specific sets of carbohydrates on foreign cells (pathogen-associated molecular patterns, PAMPs). The lectin and classical pathways converge at the cleavage of C2 and C4 by different mechanisms. In the lectin pathway, C4 and C2 cleavage occurs through mannose-binding lectin-associated serine proteases (112).

Like the classical and lectin pathways, the alternative pathway forms a C3 convertase, C5 convertase, and results in the formation of the MAC. Unlike the classical and lectin pathways, the alternative pathway may not require antibody–antigen or PAMP–PRR interactions for activation. Rather, this pathway is initiated through hydrolysis of C3 to C3(H_2_O), which is thought to occur continuously at low levels. The pathway is propagated through interactions with bacterial antigens or a lack of host surface markers (e.g., sialic acid, glycosaminoglycans, sulfated polysaccharides) (112).

Factor B, after binding C3(H_2_O), is cleaved by factor D into Ba and Bb resulting in C3(H_2_O)Bb, the fluid-phase C3 convertase (cleaves C3 to C3a and C3b). C3b binds the bacterial cell surface where it complexes with additional factor B. Factor D again cleaves factor B, which results in the second, predominant and cell-bound C3 convertase (C3bBb). This cell-bound C3 convertase is stabilized by properdin (C3bBbP). Binding of additional C3b to C3 convertase results in the formation of C5 convertase (C3bBbC3b), which cleaves C5 and initiates the formation of the MAC as described above.

## Inhibition of the Mammalian Complement System by *Borrelia* and *Ixodes*

Regulation of complement is critical for survival of host cells (122, 123). Numerous mechanisms have evolved in hosts to prevent aberrant activation of complement on host cells including the use of complement regulatory factors and host cell surface components (e.g., sialic acid). Pathogens that inhibit host complement use mechanisms that are inextricably tied to host regulatory processes. *Borrelia* use several native proteins to inhibit complement [i.e., FHBPs or complement regulator-acquiring surface proteins (CRASPs), p43, BBK32, BGA66, BGA71, CD59-like protein] (124). The following sections focus on the complement regulators factor H (FH), factor H-like protein-1 (FHL-1), factor I (FI), C4b-binding protein (C4BP), and CD59.

At least for Lyme borreliae, resistance to complement varies by strain and species (125–130). Roughly, 10% of *B. burgdorferi s.s*. are serum resistant, and 90% are intermediately resistant to serum; 75% of *B. afzelii* isolates are resistant, and 25% are intermediate; 100% of *B. garinii* isolates are sensitive (specifically, OspA serotypes 3, 5, 6, 7); *B. bavariensis* (formerly *B. garinii* OspA serotype 4) is intermediately resistant. To the best of our knowledge, similar comparisons of multiple strains and species have not been published for relapsing fever *Borrelia*, though complement resistance is not universal among relapsing fever species among the strains observed. Resistance to complement is important for the transmission, survival, and dissemination of some *Borrelia* spp. in mammalian and rodent hosts and reservoirs (131). Many *Borrelia* spp., particularly Lyme borreliae, are masters of complement evasion due to the native anticomplement proteins some possess and the ability all infectious strains possess to co-opt tick and host complement regulatory proteins.

### FH, FHL-1, and FI

Factor H is an ubiquitous 150-kDa soluble protein produced by diverse cell types throughout the human body (e.g., hepatic cells, fibroblasts, monocytes, endothelial cells) (132). FH consists of 20 short consensus repeats, while FHL-1 is a truncated variant of FH consisting of the FH N-terminal short consensus repeats 1 through 7. Both FH and FHL-1 are major direct regulators of the alternative complement pathway. In addition, FH and FHL-1 can directly regulate the classical and lectin pathways, though the regulatory roles in these pathways are minor compared to other classical and lectin regulatory mechanisms. Regulation is achieved through the recognition of self and non-self molecules *via* domains located on the C- and N-terminals, respectively (133–135). The C-terminal discriminates self from non-self through interactions with sialic acids, glycosaminoglycans, and sulfated polysaccharides, which are typically found only on host cells (136–140). FH binds self molecules with high affinity to prevent activation of complement. FH regulates the classical and lectin pathways by acting as a co-factor for FI. In this capacity, FH facilitates the serine protease activity of FI in cleaving and inactivating C3b. The alternative pathway is regulated through FH targeting factor Bb, which prevents the formation of fluid-phase C3 convertase and promotes decay (“decay acceleration activity”) of C3 and C5 convertases (141). For comprehensive reviews of FH and FHL-1, see Ref. (132, 141, 142).

### FHBPs and CRASPs

Interactions with FH are the best-studied mechanism for *Borrelia* complement inactivation, and complement resistance is correlated with binding FH (143). *Borrelia* spp. bind FH and/or FHL-1 through various native proteins collectively termed FHBPs or CRASPs (125, 144, 145). CRASPs can be grouped by their ability to bind only FH or both FH and FHL-1 as well as the species specificity of binding (that is, whether a FHBP can bind FH from only one or several host species) (125, 145): CRASP-1 (CspA) and CRASP-2 (CspZ) bind both FH and FHL-1, while CRASP-3 (ErpP), CRASP-4 (ErpC), and CRASP-5 (ErpA) bind only FH. CRASPs bind soluble FH and maintain it in an active conformation thereby allowing FH to inhibit completion of the complement response (i.e., MAC formation).

Several relapsing fever spirochetes bind FH *in vitro* (125, 146–151). Two FHBPs, FhbA and BhCRASP-1, have been identified in *B. hermsii* strains YOR and HS1, respectively (152, 153). However, binding FH is not as important for relapsing fever spirochetes to establish infection as it is for Lyme disease *Borrelia* (154, 155). Further supporting the non-essential nature of binding FH, Woodman et al. (154) found that despite FhbA being surface exposed and strongly binding FH *in vitro*, only 16% of *B. hermsii* recovered from the blood of infected mice had detectable levels of bound FH.

### C4b-Binding Protein

C4b-binding protein (C4BP) has regulatory roles in all three pathways, though is the major regulator of the classical and lectin pathways. C4BP facilitates inactivation of C4b (classical, lectin) and fluid-phase C3b (alternative) by binding C4b, displacing C2a, and facilitating FI-mediated inactivation of C3 and C5 convertases (156).

Some Lyme and relapsing fever *Borrelia* spp. bind human and various animal C4BP (143, 148, 149, 157, 158). A comprehensive analysis identified outer surface proteins (Osps) associated with C4BP including OspA, Vlps, variable membrane proteins (Vmps), and several unidentified Osps (159). However, other studies have observed no binding of C4BP by *Borrelia* spp (143, 150, 160). These contradictory data may be due to differences in experimental design including the use of different strains, growth medium, temperatures, growth phases, and the use of recombinant versus native human C4BP. A putative C4BP receptor, p43, has been identified in *B. burgdorferi s.l*. (157). The relapsing fever spirochetes *B. recurrentis* and *B. duttonii* produce CihC, a surface lipoprotein homologous in sequence and function to fibronectin-binding proteins of other relapsing fever spirochetes, which also binds C4BP (148).

### FHBP, C4BP, and *Borrelia* Niche

Resistance to complement is positively correlated to the infectivity of some *Borrelia* strains (130). With a higher resistance to complement, the more likely a bacterium can survive, disseminate, and proliferate. Co-opting tick proteins will protect spirochetes during the initial stages of transmission and dissemination but sustained dissemination requires *Borrelia* to resist complement *via* its own native mechanisms.

This leads to the question of how complement sensitive strains can cause infection. An interesting hypothesis was developed regarding complement resistance and spirochete niche when a relationship was noted between binding of the complement inhibitors, C4BP and FH (157, 158, 161). Neurotropic strains (e.g., *B. bavariensis, B. garinii, B. turicatae, B. duttonii*, and to a lesser extent *B. hermsii*) do not have to be highly resistant to complement in immunoprivileged sites, such as the central nervous system. Finding neurotropic species strongly bind C4BP and very weakly bind FH and FHL-1, while species that are not neurotropic bind C4BP but preferentially bind FH and/or FHL-1 supports this hypothesis (157). Alitalo et al. (162) did find *B. garinii* strains isolated from neuroborreliosis patients not only express FHBPs not expressed by strains cultured *in vitro* for an extended time but the FHBPs also bind FH. This implies complement resistance, though this was not reported and one of the isolates (LU59) was later reported to be highly but not completely sensitive to complement (163). It is possible that strong binding of FH is an artifact seen *in vitro*, similar to that observed with relapsing fever spirochetes (see FHBPs and CRASPs). Thus, binding FH is not required for neurotropic strains. Perhaps C4BP is sufficient to prevent complement activation during migration of neurotropic species from the site of inoculation to immunoprivileged sites. On the other hand, binding FH may be important for neurotropic strains to resist complement during migration and the incomplete sensitivity observed by Sandholm et al. (158) may be due to *in vitro* culturing resulting in the population losing its ability to bind FH. It could also be that neither C4BP nor FHBPs play a role in complement-sensitive borreliae disseminating and a novel mechanism is utilized by complement-sensitive strains.

### CD59-Like Protein

Little information is available regarding the CD59-like protein of *B. burgdorferi*. Pausa et al. (164) demonstrated an increase in serum sensitivity and MAC formation in a serum-resistant *B. burgdorferi* isolate treated with anti-CD59 antibodies compared to the control treated *B. burgdorferi* and the serum-sensitive *B. garinii* isolate. In eukaryotic cells, CD59 is a surface-exposed membrane protein that prevents C9 polymerization and thus the formation of the MAC (19, 20). Still, it is not clear *Borrelia* possesses a protein homologous to mammalian or rodent CD59. While human anti-CD59 antibodies bound a surface-exposed integral membrane protein (29 kDa), this protein has never been identified though several known borrelial proteins can and have been ruled out based on molecular weight (e.g., BGA66, BGA71, OspA, OspB, OspC) (124). Given the demonstrated complement resistance conferred by this unknown borrelial protein, more attention should be given to identifying and clarifying the role this protein plays in complement resistance.

### Complement Inhibition by *Ixodes* and *Ornithodoros* Salivary Proteins

A large number of proteins with a vast array of functions have been identified in the saliva of feeding *Ixodes* spp. with more being identified and characterized (165–169). While the details and mechanisms for some of these proteins remain to be elucidated, the beneficial nature of *Ixodes* salivary proteins to spirochete transmission and survival has been established (170–177). *Ixodes* saliva contains adaptive and innate immunomodulatory and anticomplement proteins (165, 178–183). A recent study demonstrated changes in the salivary protein profile over the course of a feeding, which has implications for the efficacy of the host immune response at the feeding pit and for transmitting spirochetes (168). Currently, several members of the anticomplement family of proteins have been characterized from *I. scapularis, I. ricinus*, and *I. persulcatus* including Salp15, Salp20, Isac, Irac I, Irac II, and Ixac-B1, -2, -3, -4, -5.

Salp15 is able to inhibit both adaptive and innate immune responses (184, 185). Salp15 binds OspC, which both serum-resistant and serum-sensitive *B. burgdorferi s.l*. produce, to inhibit deposition of the MAC and block the recognition and binding of antibodies to OspC (172, 186–188). In addition, Salp15 expression increases when ticks are infected with *B. burgdorferi* (172). Interestingly, mice passively immunized with anti-Salp15 antibodies were protected from infection with *B. burgdorferi* (189).

Salp20 inhibits the alternative complement pathway through binding properdin, which prevents stabilization of C3 convertase and propagation of the alternative pathway (183, 190, 191). In addition, Salp20 enhances the activity of FH to inhibit the alternative pathway (183). Incubating a serum-sensitive *B. garinii* strain with Salp20 protected the strain from complement activation and lysis (190). The mechanism(s) by which Salp20 confer(s) protection to *B. garinii* is unknown.

The Isac-like family of proteins include *Ixodes scapularis* anticomplement (Isac), *I. ricinus* anticomplement (Irac I), Irac II, and Ixac-B1 through -5 (*I. ricinus* anticomplement). Proteins in this family are similar in function to Salp20 (180, 182, 192). Inhibition of the alternative complement pathway is achieved through targeting C3 convertase *via* interactions with properdin, as Salp20 does, and by preventing factor B from binding C3b or displacing factor B from C3 convertase.

*Ornithodoros* salivary gland extracts also possess proteins that inhibit the host immune response (193). To date, however, one complement inhibitor has been identified and characterized from one *Ornithodoros* spp. *O. moubata*, found in Africa, is the vector of the relapsing fever spirochete *B. duttonii* (194). *O. moubata* complement inhibitor (OmCI) is a lipocalin that binds to and prevents cleavage of C5 (195, 196). OmCI was found to be effective at inhibiting C5 cleavage in different mammalian and rodent hosts (196). It is unknown if OmCI protects *B. duttonii* or if homologous proteins are found in other *Ornithodoros* spp.

## Evasion by *Borrelia* of the Mammalian Humoral Immune Response by Surface Protein Variation

Evasion of complement is undoubtedly a vital mechanism to ensure spirochetes survive and establish infection. However, *Borrelia* will elicit an humoral immune response, and there are clear roles for this immune response in controlling and preventing *Borrelia* infection (113–118, 197, 198). These responses form the basis of an intense research effort for effective Lyme vaccines. Fortunately for *Borrelia*, they are quite adept at evading the host humoral response primarily through variation of surface-exposed proteins. Lyme disease *Borrelia* possess Osps and variable membrane protein-like [Vmp-like sequences (Vls)] proteins, while relapsing fever *Borrelia* possess Vmps (includes variable large and variable small proteins) (199–203). Some species hide antigens by inducing erythrocyte rosetting (204).

### Osps and VlsE of Lyme *Borrelia*

The Osps, particularly OspC, are one of the most studied group of *Borrelia* proteins. For comprehensive reviews of *Borrelia* Osps, see Ref. (145, 205, 206). OspE and OspF are discussed above with FHBPs. Less is known about OspA, a protein predominantly involved in uptake and survival in ticks. OspA is immunogenic and able to block antibody binding to another surface-exposed protein, P66 (207, 208).

OspC has diverse roles, many of which are essential for transmission from *Ixodes* and establishing infection in mammals (209–216). These studies were key in demonstrating that *ospC* is upregulated during the early stages of infection, downregulated after infection has been established, and deleting or overexpressing *ospC* results in spirochetes that are quickly cleared from a host.

A handful of immune evasion functions have been identified for OspC. As discussed above, OspC protects *Borrelia* by binding Salp15. OspC also prevents phagocytosis by macrophages (216). In addition, several OspC types have been identified and correlated with a strains ability to establish infection in hosts and reservoirs (217–222). However, as each Osp is present as a single-copy locus, genetic variation is seen at the population level. That is, outside of random mutation or horizontal gene transfer events, a single spirochete cannot produce different OspC types *in situ*.

**Figure 2:**
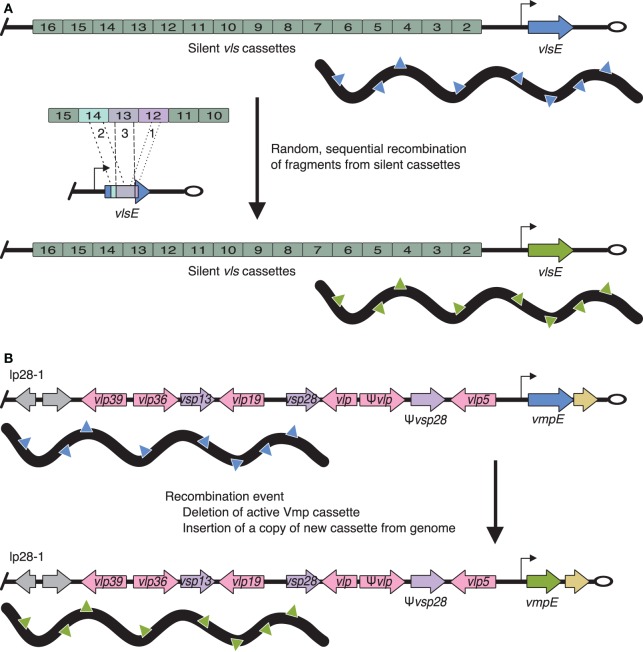
**Antigenic variation of Lyme borreliae VlsE and relapsing fever borreliae Vmp systems**. **(A)** VlsE: the expression locus (*vlsE*) is located near the telomere (open oval) of linear plasmid (lp) 28-1 (blue or green arrow, promoter is indicated by a black arrow). Silent *vls* cassettes are located upstream and in the opposite orientation of *vlsE*. Antigenic variation occurs through the random and sequential insertion of silent cassette fragments (labeled 1, 2, and 3). **(B)** *vlp* (pink arrows) and *vsp* (purple arrows) cassettes are located throughout the genome on lp28-1, 28-2, 28-3, 28-4, and 32-1. The expression locus (blue or green arrow, promoter is indicated by a black arrow) is found on lp28-1 near the telomere (open oval). Changing the expressed Vmp cassette is achieved through deletion of the current cassette (blue arrow) followed by insertion of a copy of a new cassette (green arrow *via* recombination events) resulting in a change in the expressed Vmp on the surface of the bacterium (denoted by blue or green triangles, respectively). Gray arrows indicate non-Vmp ORFs; tan arrows indicate downstream homology sequences (DHS, sequences found throughout the genome and required for mapping recombination events at the Vmp expression locus).

In contrast, the Vls system can change the expressed surface antigen *in situ* (Figure 2). Antigenic recombination of VlsE is important in maintaining infection in mammals and helps Lyme *Borrelia* evade the humoral immune response (223–236). The Vls system is composed of approximately 16 *vls* cassettes (the exact number varies by strain) and one expression locus, *vlsE*. All of the identified *vls* cassettes are located on the same plasmid (lp28-1) in close proximity to but in the opposite direction of *vlsE*. Expression at *vlsE* occurs through the random recombination of segments of multiple *vls* cassettes rather than recombination of an entire, single *vls* cassette. Thus, recombination events result in thousands of unique VlsE variants, all approximately 36 kDa.

### Vmps of Relapsing Fever *Borrelia*

Variable membrane proteins, a system similar to Vls, are one of the best characterized immune evasion mechanisms (199, 237–240). *B. hermsii* has approximately 60 unique and promoterless *vmp* cassettes (i.e., silent cassettes) scattered throughout its genome and one promoter-driven *vmp* expression locus (Figure 2). A single *vmp* cassette is expressed when the entire cassette is moved to the expression locus.

The majority of spirochetes are cleared from the host through specific anti-Vmp IgM antibodies raised against the predominantly expressed Vmps, which results in a significant decrease in spirochete load (from approximately 10^5^–10^7^to <10^4^ spirochetes/mL). The remaining spirochetes consist of a small population expressing different cassettes. Since the host has not raised a strong antibody response to these non-dominantly expressed Vmps, this minority population of spirochetes can proliferate to high concentrations until an antibody response is mounted and the majority of spirochetes are once again cleared. This cycle of *vmp* conversion, peaking spirochete loads, and antibody-mediated clearing repeats a minimum of two times resulting in the characteristic symptoms of a relapsing fever illness.

## Mechanisms of Immune Evasion by *B. miyamotoi*: Where We are

Given the genetic similarity of *B. miyamotoi* to relapsing fever spirochetes, it is likely *B. miyamotoi* utilizes some homologous mechanisms to evade host immune responses. While *B. miyamotoi* is resistant to complement *in vitro* (241, 242), complement inactivation is not required for relapsing fever spirochetes to establish infection. OspE homologs have been identified in *B. miyamotoi* FR64b (isolated from the blood of *A. argenteus*); however, McDowell et al. were unable to demonstrate FH-binding (125). This suggests, as is the case for relapsing fever spirochetes, inactivation of complement may not be required to resolve spirochetemia during infection with *B. miyamotoi* (115, 243).

Instead, it appears *B. miyamotoi* utilizes a Vmp system (244), and Wagemakers et al. (100) recently demonstrated antigenic variation of Vmps in *B. miyamotoi*. C3H/HeN mice infected with *B. miyamotoi* LB-2001 produced anti-Vsp1 IgM and IgG antibodies that were effective in clearing the initial spirochetemic peak of *B. miyamotoi* from SCID mice. Despite this clearing, a second spirochetemic relapse occurred. Analyses of the secondary *B. miyamotoi* population revealed expression of *vlpC2*, not *vsp1*, as would be expected in the case of antigenic variation. They also noted *vlpC2* was present in the initial *B. miyamotoi* population in a much lower prevalence compared to *vsp1*.

## Mechanisms of Immune Evasion by *B. miyamotoi*: Where We Need to be

Even though *B. miyamotoi* is genetically similar to relapsing fever spirochetes, it has evolved and exists in different vectors (*Ixodes* not *Ornithodoros*) with different enzootic cycles and different co-pathogens compared to relapsing fever spirochetes. We should not assume *B. miyamotoi* utilizes the same set of mechanisms as other relapsing fever spirochetes. *B. miyamotoi* may use a combination of relapsing fever and Lyme *Borrelia* mechanisms as well as completely novel mechanisms.

The role of IgM in clearing *B. miyamotoi* has not been demonstrated. As discussed above, IgM is key in clearing relapsing fever infections. During *B. hermsii* infections, IgM targets FhbA and other surface proteins (113). IgM likely is important in clearing *B. miyamotoi*. All immunocompromised patients diagnosed with a *B. miyamotoi* infection developed meningoencephalitis. A shared factor with these patients has been treatment with rituximab, a monoclonal anti-CD20 antibody targeting IgM-producing CD20-positive B cells. Depletion of B cells may explain how *B. miyamotoi* is able to migrate to the CNS and causes meningoencephalitis in patients treated with rituximab. The presence of unknown complement inhibitors, however, could contribute to the complement resistance of *B. miyamotoi* and may be useful in establishing infection (241, 242).

The effects of tick saliva on *B. miyamotoi* survival have not yet been studied. However, being vectored by *Ixodes, B. miyamotoi* likely takes advantage of the protective proteins in tick saliva. In addition, understanding interactions between host, vector, and pathogen will aid in the development of Lyme and relapsing fever prevention strategies and thus requires more attention.

## Concluding Remarks

Infection with *B. miyamotoi* in immunocompetent patients generally results in non-specific symptoms (e.g., headache, malaise), recurrent fever, and spirochetemia characteristic of relapsing fever. However, additional symptoms characteristic of relapsing fever have not been demonstrated, namely rapid symptom onset with a crisis event suggesting *B. miyamotoi* infection is not synonymous with relapsing fever and is rather a relapsing fever-like illness (90). This should not be surprising given the different lifestyle of *B. miyamotoi* compared to the vast majority of relapsing fever spirochetes.

The ability to evade the immune response is important for any successful pathogen but many of the mechanisms *B. miyamotoi* utilizes remain undiscovered. Hindered by the lack of a robust animal model, the relatively long-standing inability to culture *in vitro*, and being unaware of its pathogenicity, our understanding of *B. miyamotoi* is still in its infancy. However, we are making large strides forward with recent advances in culture techniques, animals models, physicians actively considering *B. miyamotoi* infection, as well as a growing wealth of epidemiological data that will allow us to clarify the details of infection, genetics, and physiology of this emerging pathogen.

## Author Contributions

All authors listed have made substantial, direct, and intellectual contribution to the work and approved it for publication.

## Conflict of Interest Statement

The authors declare that the research was conducted in the absence of any commercial or financial relationships that could be construed as a potential conflict of interest.
